# Diversity of *Cercopithifilaria* species in dogs from Portugal

**DOI:** 10.1186/1756-3305-7-261

**Published:** 2014-06-05

**Authors:** Helder CE Cortes, Luís Cardoso, Alessio Giannelli, Maria Stefania Latrofa, Filipe Dantas-Torres, Domenico Otranto

**Affiliations:** 1 Victor Caeiro Laboratory of Parasitology, Instituto de Ciências Agrárias e Ambientais Mediterrânicas, University of Évora, Évora, Portugal; 2 Department of Veterinary Sciences, School of Agrarian and Veterinary Sciences, University of Trás-os-Montes e Alto Douro (UTAD), Vila Real, Portugal; 3 Parasite Disease Group, Instituto de Biologia Molecular e Celular, Universidade do Porto, Oporto, Portugal; 4 Department of Veterinary Medicine, University of Bari, Valenzano, Italy; 5 Department of Immunology, Centro de Pesquisas Aggeu Magalhães (Fiocruz), Recife, Brazil

**Keywords:** *Cercopithifilaria bainae*, *Cercopithifilaria grassii*, *Cercopithifilaria* sp. II, Dogs, Portugal

## Abstract

**Background:**

Filarioids belonging to the genus *Cercopithifilaria* (Spirurida: Onchocercidae) have been described in dogs in association with *Rhipicephalus sanguineus* group ticks, which act as their biological vectors. This study represents the first investigation on *Cercopithifilaria* spp. in dogs from Portugal.

**Findings:**

Dogs (n = 102) from the Algarve region (south of Portugal) were sampled by skin snip collection and tissues were left to soak overnight in saline solution. Sediments were observed under a light microscope and the detected microfilariae identified according to their morphology. Twenty-four dogs (23.5%) were found infected with at least one species of *Cercopithifilaria*, namely *C. bainae* (9.8%), *C. grassii* (3.9%) and *Cercopithifilaria* sp. II *sensu* Otranto *et al*., 2013 (13.7%). Results were confirmed by molecular amplification of partial cytochrome *c* oxidase subunit I and 12S rRNA genes and sequence analysis. Co-infections with more than one *Cercopithifilaria* species were detected in 3.9% of the animals.

**Conclusions:**

This is the first report of *Cercopithifilaria* spp. in dogs from Portugal. The estimated level of infection with *C. bainae*, *C. grassii* and *Cercopithifilaria* sp. II suggests that these filarioids are prevalent in the canine population of southern Portugal.

## Findings

Several species of vector-borne filarial nematodes (Spirurida: Onchocercidae) have been found parasitizing dogs in Europe. In particular, *Dirofilaria immitis*, also known as the canine heartworm, and *Dirofilaria repens* are the agents of cardiopulmonary and subcutaneous dirofilarioses, respectively, which represent diseases of veterinary importance and zoonotic potential [1]. These two species, together with *Acanthocheilonema dracunculoides* and *Acanthocheilonema reconditum*, have microfilariae that circulate in the bloodstream. However, dogs may also be infected with other less well-known filarial species, such as *Onchocerca lupi* and *Cercopithifilaria* spp., whose microfilariae are found in dermal tissues [2,3].

The genus *Cercopithifilaria*, originally described as a subgenus of *Dipetalonema* by Eberhard [4], currently comprises 28 species that are parasites of a broad range of wild and domestic mammals and are transmitted by ixodid ticks [5-7]. Adult worms usually dwell beneath the cutaneous tissues, where they are not easily detected, and their microfilariae are found exclusively in the dermis [5]. Filarioids of the genus *Cercopithifilaria* have been described in dogs from Brazil and Europe [6,8]. Up until now, *Cercopithifilaria grassii*, *Cercopithifilaria bainae* and *Cercopithifilaria* sp. II *sensu* Otranto *et al.*, 2013 have been morphologically and molecularly differentiated in canine populations in Europe [7,9-11]. Microfilariae of these three species can be distinguished from each other by their length and general morphological features and also by the inter-specific genetic distance of the mitochondrial cytochrome *c* oxidase subunit I (*cox*1) and 12S rRNA genes [6,10].

The field distribution of *C. bainae* overlaps with that of *Rhipicephalus sanguineus* group ticks [12], which act as vectors for this nematode [2], as experimentally demonstrated [13,14]. *C. bainae* has been found in dogs and ticks from Italy, Spain and Greece [2,15], in one dog from Romania [11] and also in ticks from Portugal [16]; M.S. Latrofa, F. Dantas-Torres, A. Giannelli and D. Otranto, personal communication.

Infections with canine filarioids, such as *D. immitis*, *A. dracunculoides*, *A. reconditum* and *O. lupi*, have previously been identified in Portugal [17-19], but no information on *Cercopithifilaria* spp. is available from dogs. In order to fill this gap of knowledge, in November 2012, 102 dogs (65 females and 37 males) housed in two shelters located in center-eastern areas of the Algarve region (the southernmost part of mainland Portugal) were sampled. The animals were estimated to be 1-3 years (n = 19), 4-8 years (n = 78), 9-11 years (n = 4) or 12-15 years old (n = 1). An informed consent for inclusion of dogs in this survey was obtained from the owner before sampling. The study design and experimental procedures were approved by the ethical commission of the University of Évora as complying with the Portuguese legislation for the protection of animals (Law no. 92/1995). Skin snips of around 0.01 cm^3^ (0.2 × 0.2 × 0.2 cm) were collected from the forehead region using a disposable scalpel and soaked in saline solution (pH 7.4) for 12 h in an incubator at 30°C. Dermal microfilariae were searched for in 20 μl sediment placed on a glass slide, using a light microscope (magnifications of ×40 and ×100), and identified according to their morphological features, as previously described [6,10]. Microfilariae were photographed with a digital camera mounted on the microscope (DP20, Olympus, Japan) and measurements (in micrometers) taken with the Live Cell Imaging software (Olympus).

Following morphological analysis of microfilariae, sediments scoring positive were stored in 70% ethanol and species identification was subsequently assessed by PCR amplification and DNA sequencing [6,10]. Microfilariae were isolated and genomic DNA extracted from individual specimens using a commercial kit (DNeasy Blood & Tissue Kit, Qiagen, Germany) in accordance with the manufacturer’s instructions. Samples were molecularly processed for specific amplification of *cox*1 and 12S rRNA gene fragments (∼689 and ∼330 bp in size, respectively), with primers and procedures as described elsewhere [6].

Amplicons were purified using Ultrafree-DA columns (Amicon, Millipore, USA) and sequenced directly with the Taq DyeDeoxyTerminator Cycle Sequencing Kit (v.2, Applied Biosystems, USA) in an automated sequencer (ABI-PRISM 377, Applied Biosystems). The sequences were determined from both strands, using the same primers individually as for PCR, and the electropherograms verified by eye. The sequences were aligned using ClustalW program and compared among them and with those available in GenBank dataset by Basic Local Alignment Search Tool (BLAST - http://blast.ncbi.nlm.nih.gov/Blast.cgi).

For statistical analysis, the Chi-squared or Fisher’s exact tests compared proportions of positivity and a *p* value < 0.05 was considered as statistically significant [20]. The exact binomial test estimated confidence intervals (CI) for proportions, with a 95% confidence level. Analyses were done using StatLib or SPSS 21 software for Windows.

**Figure 1:**
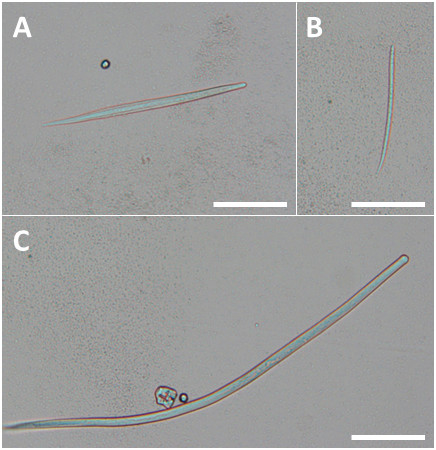
**Microfilariae of: (A) *Cercopithifilaria *sp. II; (B) *Cercopithifilaria bainae*; and (C) *Cercopithifilaria grassii.*** Sediment of forehead skin snips from dogs (scale-bar = 100 μm).

By microscopy, microfilariae of *C. bainae*, *C. grassii* and *Cercopithifilaria* sp. II were detected (Figure 1), with an individual prevalence of 9.8% (CI: 4.8-17.3%), 3.9% (CI: 1.1-9.7%) and 13.7% (CI: 7.0-20.8%), respectively, both in single or in mixed infections. The prevalence of *Cercopithifilaria* sp. II was significantly higher than that of *C. grassii* (*p* = 0.023), but no differences were found in pairwise comparison between *C. bainae* and *C. grassii* or *C. bainae* and *Cercopithifilaria* sp. II. The prevalence of single infections (19.6%) was significantly higher (*p* < 0.001) than that of mixed infections (3.9%) (Table 1). Differences in the prevalence of infection with at least one of the three *Cercopithifilaria* species were not statistically significant between female (24.6%) and male dogs (21.6%) or between age groups, i.e. 1-3 years (31.6%), 4-8 years (21.8%), 9-11 years (25.0%) and 12-15 years (0.0%).

**Table 1:** **Prevalence of single and mixed infections with *Cercopithifilaria *** **species as determined by morphological identification of microfilariae in 102 dogs from the Algarve (south of Portugal)**

***Cercopithifilaria *** **spp.**	**No. of positive dogs**	**%**	**CI (%)**
Single infections	20	19.6^a^	12.4–28.6
*Cercopithifilaria bainae*	7	6.9	2.4–11.6
*Cercopithifilaria grassii*	3	2.9	0.6–8.3
*Cercopithifilaria* sp. II	10	9.8	4.8–17.3
Mixed infections	4	3.9^a^	1.1–9.7
*C. bainae* + *Cercopithifilaria* sp. II	3	2.9	0.6–8.3
*C. grassii* + *Cercopithifilaria* sp. II	1	1.0	0.02–5.3
Total	24	23.5	15.7–33.0

CI: confidence interval; ^a^: *p* < 0.001.

Microfilariae of *C. bainae* (n = 10) had an average length ± standard deviation (SD) of 181.1 ± 7.5 μm (range: 170.1-196.4 μm). The average length ± SD of *C. grassii* microfilariae (n = 6) was 630.8 ± 13.8 μm (range: 610.1-643.5 μm). Microfilariae of *Cercopithifilaria* sp. II (n = 13) had an average length ± SD of 277.0 ± 15.5 μm (range: 260.9-307.4 μm).

The BLAST analysis revealed 99% homology of the amplified sequences with the closest accession numbers of *C. bainae* (JF461457 and JF461461), *C. grassii* (JQ837810 and JQ837812) and *Cercopithifilaria* sp. II (JQ837809 and JQ837811) available in GenBank database (accessions displayed for *cox*1 and 12S rRNA genes, respectively).

Morphological and molecular data concurred in indicating that the dermal microfilariae detected in dogs from southern Portugal belong to three distinct known species of *Cercopithifilaria*. Infections with *C. bainae* and *Cercopithifilaria* sp. II were detected in dogs from the Italian regions of Basilicata [10] and Sardinia [15], whereas *C. bainae* and *C. grassii* were detected in Sicily [10]. Sympatric *C. bainae*, *C. grassi* and *Cercopithifilaria* sp. II populations were found in dogs from central Spain [10]. The 23.5% overall prevalence of infection with at least one of those species (Table 1) is in line with that of 21.6% found by microscopy in dogs from central Spain [10]. In Italy, the prevalence of *Cercopithifilaria* spp. has been found to range from 9.4% in Sardinia [15] to 13.3% in Sicily [10]. Still by microscopic examination of skin snip sediments, the prevalence of *C. bainae* in northeastern Greece was 4.3% [2].

Dogs found infected with *Cercopithifilaria* spp. did not present physical abnormalities that could be associated to the presence of microfilariae in the skin (data not shown). However, based on clinical and histological findings, a pruritic, diffuse and erythematous dermatitis without any other apparent cause was previously described in two dogs infected with microfilariae of *C. bainae*[21] or *Cercopithifilaria* sp. II [10].

In conclusion, the present study highlights that *Cercopithifilaria* spp. should be considered among those parasites that can cause dermal microfilariae infection in dogs in Portugal. In the meantime, further studies should be carried out in order to elucidate the pathogenic relevance of these filarioids for dogs and other potential hosts.

## Competing interests

The authors declare that they have no competing interests.

## Authors’ contributions

HCEC designed and supervised the study, collected samples, analyzed data, assisted in drafting and reviewed the manuscript; LC collected samples, analyzed data and wrote the manuscript; AG performed morphometric assessments and reviewed the manuscript; MSL performed DNA extraction and molecular analyses and reviewed the manuscript; FDT analyzed data and reviewed the manuscript; DO planned and supervised the study, analyzed data and reviewed the manuscript. All authors read and approved the final manuscript.
